# Endocrine Disruptors Leading to Obesity and Related Diseases

**DOI:** 10.3390/ijerph14101282

**Published:** 2017-10-24

**Authors:** Demetrios Petrakis, Loukia Vassilopoulou, Charalampos Mamoulakis, Christos Psycharakis, Aliki Anifantaki, Stavros Sifakis, Anca Oana Docea, John Tsiaoussis, Antonios Makrigiannakis, Aristides M. Tsatsakis

**Affiliations:** 1 Department of Forensic Sciences and Toxicology, Faculty of Medicine, University of Crete, 71003 Heraklion, Crete, Greece; loukia.vassilopoulou@gmail.com (L.V.); tsatsaka@uoc.gr (A.M.T.); 2 Department of Urology, University General Hospital of Heraklion, Medical School, University of Crete, 71003 Heraklion, Crete, Greece; 3 Department of Obstetrics and Gynecology, Venizeleio—Pananio General Hospital of Heraklion, 71409 Heraklion, Crete, Greece; chrispsycharakis@hotmail.com; 4 Crete Fertility Center, 56, Arch. Makariou & Sof. Venizelou Str., 71202 Heraklion, Crete, Greece; alikianifantaki@gmail.com; 5 Mitera Hospital, 71409 Heraklion, Crete, Greece; stavros.sifakis@yahoo.com; 6 Department of Toxicology, Faculty of Pharmacy, University of Medicine and Pharmacy, Petru Rares, 200349 Craiova, Romania; ancadocea@gmail.com; 7 Laboratory of Anatomy-Histology-Embryology, Medical School, University of Crete, 71003 Heraklion, Crete, Greece; jtsiaoussis@hotmail.com; 8 Department of Obstetrics and Gynecology, Medical School, University of Crete, 71003 Heraklion, Crete, Greece; makrygia@uoc.gr

**Keywords:** cardiovascular diseases, carcinogenesis, diabetes mellitus, endocrine disruptors, infertility, metabolic syndrome, obesity, pesticides

## Abstract

The review aims to comprehensively present the impact of exposure to endocrine disruptors (EDs) in relation to the clinical manifestation of obesity and related diseases, including diabetes mellitus, metabolic syndrome, cardiovascular diseases, carcinogenesis and infertility. EDs are strong participants in the obesity epidemic scenery by interfering with cellular morphological and biochemical processes; by inducing inflammatory responses; and by presenting transcriptional and oncogenic activity. Obesity and lipotoxicity enhancement occur through reprogramming and/or remodeling of germline epigenome by exposure to EDs. Specific population groups are vulnerable to ED exposure due to current dietary and environmental conditions. Obesity, morbidity and carcinogenicity induced by ED exposure are an evolving reality. Therefore, a new collective strategic approach is deemed essential, for the reappraisal of current global conditions pertaining to energy management.

## 1. Introduction

Obesity is defined as aberrant accumulation of body fat, often exceeding 20% of an individual’s body weight. It has received epidemic dimensions with exponentially rising prevalence nowadays [1]. Since 1980, the prevalence has more than doubled globally [2]. It is estimated that over 600 million adults are currently obese (body mass index ≥ 30 kg/m^2^); that around 40 million children prior to five years of age are overweight/obese; and that 80% of obese children remain obese in adulthood [3]. Obesity is linked to a broad spectrum of pathologies, including insulin resistance, diabetes mellitus, metabolic syndrome, cardiovascular diseases, carcinogenesis and infertility [1]. Endocrine disruptors (EDs) are compounds of exogenous origin that exert various endocrine functions in specific doses, including hormonal synthesis/transportation and adverse health effects in an organism and/or descendants [4]. Exposure may occur via placental permeation, breast milk secretion, inhalation, ingestion, and transdermal absorption modifying steroid hormone metabolism and balance [5] by altering synthesis and/or breakdown of testosterone, follicle stimulation hormone (FSH), luteinizing hormone (LH), or other hormones involved in gamete physiology, fertility, implantation, fetal morphogenesis, pregnancy outcome and post birth diseases [6]. Industrial products, pesticides, laminators and many plastic products of everyday use, flame retardants, phytoestrogens and several heavy metals may act as EDs. High production volume chemicals are also known or suspected EDs. The metabolic rate of EDs is particularly low due to prolonged half-lives. By mimicking hormonal actions, EDs exhibit a wide range of effects. Pesticides for example exhibit estrogenic [7], antiandrogenic [8], weakly stimulated aromatase activity as estrogen receptor alpha (ERα) activators [9], estrogen agonists [10,11] or androgen antagonists [12,13]. The different ways with which pesticides potentially disturb the functions of sex hormones are presented in Table 1 [11]. A big concern is related to the fact that exposure to pesticides usually occurs in mixtures; thus, synergism/potentiation effects can appear [14]. Environmental disruptions during critical windows of development can lead to subtle changes in gene expression, tissue or other levels of biological organization that lead to permanent dysfunction, resulting in increased susceptibility to obesity and other related chronic diseases such as diabetes, metabolic syndrome, cardiovascular diseases, hepatic steatosis, various types of cancer and infertility [15,16,17,18].

## 2. Endocrine Disruptorss in Obesity Induction

Endocrine disruptors may act directly or indirectly as obesogens, by promoting adipogenesis through control/fostering of lipid accumulation [19] or through energy balance; the latter is accomplished through shifting towards calorie storage by modifying basal metabolic rate, altering gut microbiota [20] promoting food storage, and altering hormonal control [21] of appetite and satiety [22]. Novel obesogenic EDs are identified at a rising rate nowadays [23], promoting lipid metabolism [24], diabetes and fatty liver [25].

The lipid tissue is a type of connective tissue, comprising of precursor and mature adipocytes, endothelial cells, cells of stromal vascular fraction, vascular smooth muscle cells, macrophages and inoblasts. It primarily acts as an energy storage compartment, where triglycerides are stowed in adipocyte aggregates potentially utilized for body response in low temperatures, hunger/fasting conditions and intense muscular exertion. However, adipose tissue exerts a significant impact on homeostasis as well, acting as a potent biosynthetic machinery of factors exhibiting endocrine function. The adipose tissue cells are capable of producing various endocrine molecules (lipokines), which include cytokines (tumor necrosis factor-1 (TNF-α) and interleukin-6 (IL-6)), prostaglandins (PGs), chemokines, proteins of the alternative complement pathway (adipsin, C3 and B), angiotensinogen, factors participating in glucose homeostasis (retinol binding protein (RBP) and adiponectin), in angiogenesis (vascular endothelial growth factor (VEGF)) and in hemostasis (plasminogen activator inhibitor protein-1 (PAI-1)) as well as other molecules (e.g., leptin, visfatin, resistin and apelin, glucocorticoids and free fatty acids).

Dysregulation of the above-mentioned pathways leads to obesity. Adipose tissue macrophage accumulation is responsible for adipose TNF-α, NOS2 (nitric oxide synthase 2) and IL-6 expression [26,27]. A strong association has been reported among the extent of infiltrating adipose tissue macrophages, other pro-inflammatory immune cells and several pro-inflammatory mediators (TNF-α, IL-1β, and IL-6), which are enhanced in obesity [28]. TNF-α levels increase due to excess secretion by macrophages residing in the adipose tissue. Lipolysis is induced, resulting in elevated IL-6 levels, while adiponectin is decreased. IL-6 is involved in systematic inflammation, fostering hepatic expression of C-reactive protein and acute phase proteins, hindering insulin action in muscle and liver cells. Chemokines display a similar pattern; as they increase in obesity, they aggravate this pathology further. Adipsin interacts with C3 and B factors, inducing triglyceride storage through esterification of fatty acids and inhibition of lipolysis. Adiponectin levels are inadequate in obesity, while leptin (an inflammatory lipokine) augments TNF-α and IL-6 production.

The biochemical role of EDs in obesity has been extensively investigated. Various xenobiotics have been shown to promote obesity [1]. Fungicides such as vinclozolin and genistein have been reported to intervene in 3T3-L1 cellular line differentiation into adipose cells [29]. Bisphenol A (BPA) exposure during pregnancy has been reported to aggregate hepatic triglycerides in descendants [30]. Organochlorines (OCs) promote adipocyte differentiation through expression of fatty acid binding protein 4 and sterol regulatory element-binding protein-1c, inducing upregulation of leptin and fatty acid synthase enhancing adipogenesis/intracellular lipid accumulation in pre-adipocytes via up-regulation of molecules responsible for lipid storage [31]. They also affect adipocyte differentiation, inducing the activities of growth factor (bone morphogenetic proteins (BMPs), epidermal growth factor receptor (EGFR), and insulin-like growth factor 1 (IGF-1)) to mesenchymal stem cells that may affect fibroblast differentiation to pre- and mature adipocytes [32].

Molecular players in obesity-induced inflammation include inhibitor of nuclear factor κ-B kinase subunit beta (IKKβ)/nuclear factor κB (NF-κB) pathway [33], Jun N-terminal kinases [34], inflammasome pathway through pregnane X receptor (PXR) activation [35], cyclooxygenase-2, B-cell lymphoma-extra large (Bcl-xL) [36], cytokines, and inducible nitric oxide synthase (iNOS) [37]. Endocrine disruptors act on α-cell biology [38], on glucagon and insulin production [39], on ATP/ADP ratio, energy balance [40], and glucose stimulated insulin secretion. 2,3,7,8-Tetrachlorodibenzodioxin (TCDD) increases notably ghrelin and glucagon, links these hormones to the wasting syndrome [41], and induces lipolysis through IGF-1, fibroblast growth factor 21 and aryl hydrocarbon receptor (AHR) Dichlorodiphenyltrichloroethane (DDT) [41] and triphenyltin [42] affect both glucose stimulated insulin secretion, as well as insulin secretion due to defective cAMP-dependent cytoplasmic Na^+^ concentration in β-cells. Aroclor 1254, a polychlorinated biphenyl (PBC) mixture (non-coplanar congeners) induces higher insulin secretion in culture media [43]. Hexabromocyclododecane down-regulates glycolysis and β-oxidation of long-chain fatty acids, leading to a reduction of ATP production and total phospholipid level increase through remodeling of phospholipids from higher free fatty acids levels, enhancing obesity/diabetes [44].

Prenatally exposed individuals to decreased perfluorooctanoic acid (PFOA) levels [45] show decreased birth weight, with subsequently elevated adipose mass/body weight after puberty [46]. Interestingly, pregnant women employed in professions categorized as possibly/probably exposed to EDs present a notable high risk of delivering low body weight newborns [47]. Rates of overweight are increasing in infants less than six months of age, meaning that a different environment in utero or postnatally exists, which has an impact on fat deposition in early life [48]. Prenatal exposure to polychlorinated biphenyls (PCBs) and dichlorodiphenyldichloroethane (DDE) may cause permanent defects predisposing to obesity later in life [49,50]. Aklylphenols through glycerol-3-phosphate dehydrogenase activity and peroxisome proliferator activation of receptor γ (PPARγ) activation [51], phthalates, organotins tributyltin-triphenyltin, phthalate metabolites, brominated flame retardants [52], polybrominated diphenyl ethers, tetrabromobisphenol A (TBBPA) and mono-(2-ethylhexyl)tetrabromophthalate (METBP) [53], increase visceral fat, accumulate adipocytes in bones, resulting in lower bone density, osteoporosis and increased fracture risk through PPARγ, which regulates the balance between adipogenesis and osteogenesis [54]. Fetal/perinatal exposure to obesogenic phthalates [55,56] and perfluorinated chemicals (PFCs) intercepts transcriptional regulation of testicular steroidogenesis, reducing androgen biosynthesis and androgen activity [57]. Low birth weight is a EDs-dependent result hailing from poor nutritional uterine environment and additively is a risk factor for obesity [58] and metabolic syndrome [59].

## 3. Endocrine Disruptors—Role in Obesity Related Diseases

### 3.1. Endocrine Disruptors Involvement in Metabolic Syndrome

Endocrine disruptors contribute to the manifestation of metabolic syndrome through inflammatory processes via cytokines/adipokines, producing the effects of metabolic imbalance. Metabolic syndrome refers to central (abdominal) obesity, insulin resistance/glucose intolerance, dyslipidemia/oxidized low-density lipoprotein (LDL), increased triglyceride/reduced high-density lipoprotein (HDL) levels, elevated blood pressure, endothelial dysfunctions and atherogenesis. Risk factors include obesity, diabetes, hypertension, decreased HDL and elevated triglyceride levels, as well as hypercoagulable states. Leptin behaves as a blood pressure regulator by activating sympathetic nervous system inducing nitric oxide (NO) production. When an adipocyte surplus exists, PAI-1 leads to thrombosis/atheromatosis. Apelin has been shown to cause angiogenesis [60] as well as angiotensinogen, with the latter presenting also as a factor resulting in atheromatosis. Important role in metabolic syndrome is attributed to TNF-α. TNF-α increased levels occurring in obesity, when combined with reduced adiponectin expression trigger NF-κB activation, which in turn enhances reactive oxygen species (ROS) adhesion molecules and cytokines expression, resulting in excess glucose, free fatty acids and incapacitated insulin sensitivity. Endorcine disruptors intervene on intermediary metabolism (Table 2) [61] through several pathways [62,63,64,65]. For example, TCDD reduces expression of lipoprotein lipase promoting hypertriglyceridemia, inhibiting glucose transport/LPL activity and increasing secretion of TNF [66]. Many of these pathways are presented herein (see Section 2, Section 3.2, Section 3.3 and Section 3.4, referring to the components of metabolic syndrome).

### 3.2. Endocrine Disruptors and Insulin Resistance

**Figure 1:**
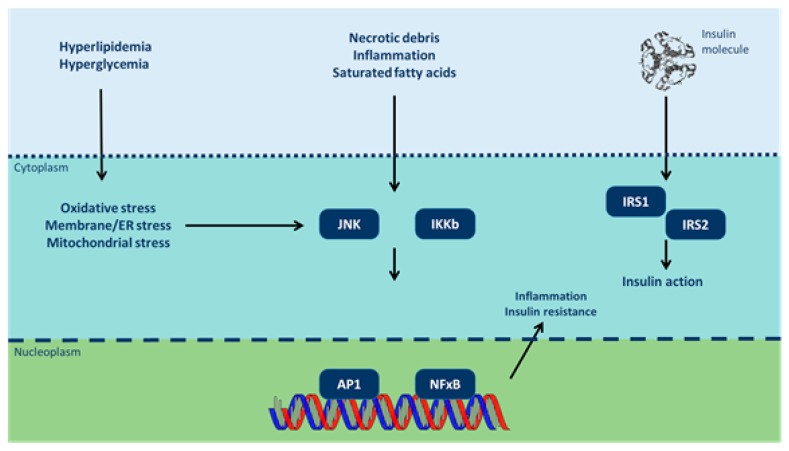
Inflammatory signaling pathways link nutrient excess to insulin resistance (modified from [67]). Cytoplasmic/nuclear responses via tyrosine phosphorylation of insulin receptor substrate (IRS)-1 and IRS-2 are activated by the presence of insulin at the cell surface. However, insulin signaling is potentially inhibited by serine phosphorylation of these proteins by Jun N-terminal kinases (JNK) and inhibitor of nuclear factor κB (NF-κB) kinases (IKK). Various intra/extracellular sequelae of chronic nutrient excess activate these signaling pathways, linking overfeeding to insulin resistance. JNK and IKK activation triggers inflammatory cytokine production, activating JNK/IKK in an autocrine/paracrine manner further reinforcing insulin resistance. ER: endoplasmic reticulum; AP-1: activator protein-1).

Insulin resistance can be provoked by various molecular patterns encountered in obesogenic events (Figure 1) [67]. TNF-α reduces insulin sensitivity by restricting glucose transporter type 4 (GLUT4) function [68]. Leptin regulates intracellular lipid levels in hepatic and β-pancreatic cells, improving insulin sensitivity [69]. On the contrary, resistin promotes insulin tolerance via TNF-α and IL-6 activation [70]. Visfatin mimics insulin and fosters the transformation of pre-adipocytes to mature adipocytes [71]. Adipsin-C3/B interaction inhibits lipolysis and glucose transportation [72]. Moreover, RBP-4 (retinol binding protein-4) excessively expressed in abnormal adipose tissue, inhibits GLUT4 expression leading to insulin resistance [73]. As insulin resistance occurs, there is an increase in fasting glucose and hindered glucose tolerance. This metabolic state induces further insulin release, ultimately resulting in hyperinsulinemia, which simulates transcription factors in the liver, driving hypertriglyceridemia and hepatic steatosis [74].

Females exposed to diethylhexyl phthalate throughout gestation/perinatal development exhibit hyperglycemia with reduced insulin levels [75]. Perinatal exposure to BPA increases the severity of insulitis (inflammation of the islets of Langerhans) and thus diabetes prevalence [76]. Endocrine disruptors induce genome alterations in pregnancy or early life and enchain in a decreased expression of pancreatic/duodenal homeobox 1 transcription factor gene (*PDX-1*)/increase of type 2 diabetes [77], suggesting that in utero exposure to under- or over-nutrition is a risk for obesity and diabetes progression in adulthood. Endocrine disruptors confine the stock of essential metabolic substrates to the fetus and cause an intrauterine growth retardation, presenting as fetal starvation and the metabolic basis that triggers diabetes progression in PDX-1. Prenatal and early life exposure to pollutants such as phthalates, BPA, perfluorinated compounds, PCBs and dioxins may negatively affect the development of immune system, resulting, among other, in type 1 diabetes mellitus via hindered pancreatic β-cell and immune-cell functions as well as immunomodulation, through hormonal and/or epigenetic alterations [78,79].

Endocrine disruptors deregulate pancreatic islet β-cell function, development of peripheral insulin resistance, insulin production, β-cell mass (compensatory hyperplasia/hypertrophy of β-cells) and impaired insulin output, insulin signaling, increasing β-cell apoptosis. Endocrine disruptors promote, by these ways, the onset of diabetes in obese insulin resistant type 2 diabetes [80]. For example, PCB induced pre-proinsulin expression via AHR activation and inhibition of transcription factor Nrf2a [81] and PFOA increase significantly the proinsulin/insulin ratio [82]. Endocrine disruptors augment the risk of diabetes through modulation of glucose metabolism [83]. Organochlorines (OCs) and PCBs act through mitochondrial dysfunction and endocrine-disrupting mechanisms [84], including PCBs effects on pancreatic β-cell function [85] and OCs adiponectin release [31]. Endocrine disruptors decrease GLP-1R (Glucagon-like peptide 1 receptor) activation by the endocrine cells of the intestine following ingestion of food, and decrease the signaling on insulin secretion by decreased number of pancreatic islet β-cells. The decreased GLP-1R increases release of pancreatic glucagon via hypothalamic receptors as a lack of satiety during eating [86]. Genes, key regulators of weight homeostasis, steroid hormone functions, insulin signaling and incretin receptor expression levels in β-cells occupy central positions, affecting the adrenal axis and β-cell glucose toxicity [87].

Endocrine disruptors play a key role in obesity-associated insulin resistance due to activation of extracellular matrix (ECM) receptor pathways in adipose tissue that constitutes the cell microenvironment [88]. Endocrine disruptors involved in ECM remodeling through ECM receptors such as integrins and CD44 contribute to inflammation, apoptosis, and angiogenesis in adipose tissue as well as skeletal muscle and liver [89]. Since excessive ECM deposition results in adipose tissue fibrosis overpassing angiogenesis capability in tissues, repressed expression of genes essential for adipose angiogenesis (e.g., *VEGFa*) appear to be mediated by activation of ECM receptor and HIF1a/VEGFa pathways [90]. For example, PCBs increase the fibroblast adhesion through type I collagen increase, implicating OC exposure as etiologic factor in a broad range of human diseases characterized by fibrosis [91]. EDs are not only restricted to ligand-binding but can also be modulated by other signaling pathways [92], including EGF, IGF, integrins (fibronectin), phosphatidylinositol 3-kinase/AKT, X-box binding protein 1 (XBP1), second messengers cAMP and dopamine; influence estrogen receptor (ER) transcriptional activity by targeting the receptor directly or by regulating co-regulators [93]. Dichlorodiphenyltrichloroethane (DDT) induces human ER, promoting gene expression (matrix metalloproteinase-2 and -9; telomerase reverse transcriptase) and inhibiting invasion-inhibited genes (tissue inhibitor of metalloproteinase-1 and -4) [94].

Prenatal/early life exposure to pollutants (phthalates, BPA, perfluorinated compounds, PCBs and dioxins) may negatively affect immune system development, resulting in in type 1 diabetes mellitus via impaired pancreatic β-cell/immune-cell functions and immunomodulation through hormonal and/or epigenetic alterations, among others [78,79]. Endocrine disruptors affect quantitative insulin secretion and immunity but also alter insulin-dependent mRNA stability. Because insulin-like growth factor-binding protein gene (*IGFBP-1*) promoter regulates blood glucose levels, the specifically up-regulation of IGFBP-1 mRNA in human hepatocytes and HepG2 human hepatoma cells (2.5- and 8-fold, respectively), even in the presence of insulin, might account for the disruptive effects of TCDD on glucose metabolism [95].

### 3.3. Endocrine Disruptors Involvement in the Manifestation of Cardiovascular Diseases

Reactive oxygen species and other oxidative stress biomarkers generated by EDs (Table 3) impair eNOS and other anti-atherosclerotic enzymes [96], triggering proatherosclerotic events by afflicting vascular tone [97]. Monocytes infiltrate vascular wall and macrophages gather oxidized low density lipoproteins leading to foam cells formation [98]. T-lymphocyte attraction, macrophage-smooth muscle cell proliferation, and collagen accumulation is provoked, leading to plaque formation. Dyslipidemia, obesity and pro-inflammatory cytokines augment the phagocytic-like NADPH oxidase expression in β-cells [99]. EDs induce endothelium-dependent arterial contractions via angiotensin II expression [100], and transcriptional down-regulation of mitochondrial uncoupling natural antioxidant [101]. Endocrine disruptors affect Notch/Wnts signaling pathways [101,102] and T-box/Homeobox genes that are strongly associated with cardiovascular diseases. 2,3,7,8-Tetrachlorodibenzodioxin exposure may result in clinically life-menacing ventricular arrhythmias [103] and polymorphic ventricular tachycardia [104], through intracellular Ca^2+^ influx by induction of early after-depolarizations occurring during action potential [105]. Perfluorooctanoic acid(PFOA)may induce a long QT interval through PPARα/BMP2 signaling pathways in early developmental stage. Polychlorinated biphenyls (PCBs)can exert negative impact in cardiac electrophysiology, resulting in toxic effects [106]. Acute endosulfan poisoning affects ventricle repolarization by impairing outward K^+^ currents or by stimulation of endogenous catecholamine secretion [107].

### 3.4. Endocrine Disruptors Involvement in Hepatic Steatosis

Highly halogenated environmental chemicals such as OCs (mirex, chlordecone, and chlordane) and persistent organic pollutants induce steatosis [108]. Chronic inflammation, pathological fibrosis, and liver branching deformities are mechanisms contributing to prolonged hepatic steatosis/exacerbated inflammation in non-alcoholic steatohepatitis [109] or non-alcoholic fatty liver disease [110] in adults [111] and children [112]. Lindane interacts with ERβ [113], increasing serum total cholesterol, triglycerides, urea, creatinine, total bilirubin, uric acid, aminotransferases, phosphatases and lactate dehydrogenase activities, while decreasing high-density lipoprotein [114]. 2,3,7,8-Tetrachlorodibenzodioxin (TCDD) has been shown to cause hepatic steatosis by epigenomic-induced ECM reprogramming and Homeobox-Tbox remodeling beginning during endometrial life or preconseptionally [115]. Non-alcoholic fatty liver disease/non-alcoholic steatohepatitis are characterized by excessive triglyceride accumulation in hepatocytes, or steatosis. 2,3,7,8-Tetrachlorodibenzodioxin has been shown to exacerbate development of hepatic fibrosis and inflammation through TCDD-induced activation of aryl hydrocarbon receptor on the regulation of ECM matrisome, e.g., synthesis, deposition, and breakdown [109]. Collagen synthesis, ECM metabolism, plasminogen activator/plasmin system, and ECM homeostasis in liver are targeted and remodeled by TCDD, in combination with TCDD effects on innate/acquired immunity mechanisms such as cytokine production/release, inflammasome activation, and gut dysbiosis [116].

### 3.5. Endocrine Disruptors Involvement in Carcinogenesis

**Figure 2:**
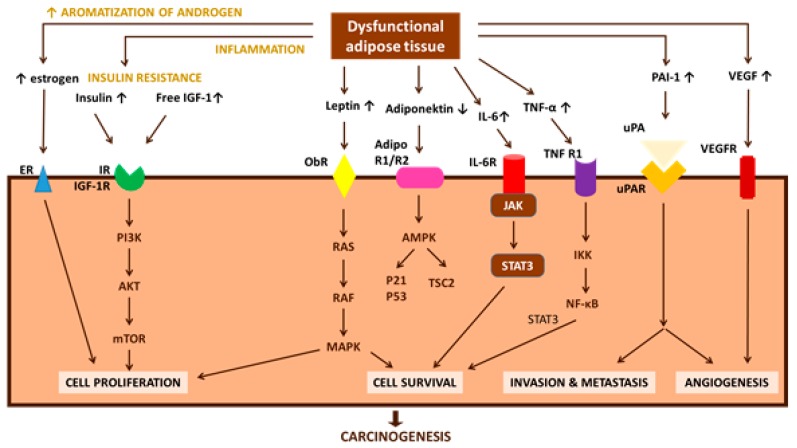
Obesity-associated changes in the physiological function of adipose tissue, which can lead to insulin resistance, chronic inflammation, and altered secretion of adipokines (direct pathogenic factors), are speculated to be involved in carcinogenesis and cancer progression (modified from [120]). AdipoR1/R2: adiponectin receptor 1/2; AMPK: 5′-AMP activated protein kinase; IGF-1: insulin-like growth factor-1; IGF-1R: insulin-like growth factor-1 receptor; IKK: IκB kinase; IL-6: interleukin-6; IL-6R: interleukin-6 receptor; IR: insulin receptor; IRS-1: insulin receptor substrate-1; JAK: Janus kinase; MAPK: mitogen-activated-protein-kinase; mTOR: mammalian target of rapamycin; NF-κB: nuclear factor-κB; ObR: leptin receptor; PAI-1: plasminogen activator inhibitor-1; PI3-K: phosphatidylinositol 3-kinase; ROS: reactive oxygen species; STAT3: signal transducer and activator of transcription 3; TNF-α: tumor necrosis factor-α; TNF-R1: tumor necrosis factor-receptor 1; TSC2: tuberous sclerosis complex 2; uPA: urokinase-type plasminogen activator; uPAR: urokinase-type plasminogen activator receptor; VEGF: vascular endothelial growth factor; VEGFR: vascular endothelial growth factor receptor.

Endocrine disruptors may cause aberrant mitogenic signal transduction in a number of ways, most often by: (i) altering protein phosphorylation (Ca^2+^, PKC, mitogen-activated protein kinase (MAPK) or c-Jun pathway). Methoxyclor [117] and nutritional supplies [118] induce epigenetic mechanisms by this way. Women with polycystic ovary syndrome present numerous transcriptional and epigenetic changes in adipose tissue that are similar to EDs effects; (ii) disrupting normal protein–protein interactions establishing abnormal ones; and (iii) altering synthesis or degradation of signaling proteins promoting mitosis and tumor formation. The contribution of dysfunctional adipocytes due to their exposure to carcinogenic EDs is important and achieved through a network of pathways. Novel adipocytokines (apelin, endotrophin, Fatty acid binding protein 4 (FABP4), lipocalin 2, omentin-1, visfatin, chemerin, angiopoietin protein 2 (ANGPTL2), and osteopontin) have been identified in various tumor types, enriching the list of potential carcinogenic factors [119]. Possible pathways directly linking obesity-associated dysfunctional adipose tissue with cancer are presented in Figure 2 [120].

Endocrine disruptors through ER-dependent increase expression of growth promoting genes responsible for cell proliferation, differentiation, and apoptosis at terminal end buds and lead to ductal elongation in the mammary gland [121] are involved in mammary tumorigenesis [122]. Organochlorines are related to an elevation in hepatocellular carcinoma rates among males [123]. There is increased pancreatic cancer mortality among habitants in areas with increased application rates of 1,3-dichloropropene (EPA-classified as probable human carcinogen), captafol, pentacholoronitrobenzene, and dieldrin [124]. Exposure to PCBs, dioxins, and furans is a known risk factor for non-Hodgkin’s lymphoma, through adverse effects on endocrine but also on immune, reproductive and neurobehavioral functions [125]. Occupational and ambient exposure to pesticides, interacting with free testosterone bioavailability and its androgen receptor (AR) binding, might be present an association to risk of prostate cancer manifestation.

**Figure 3:**
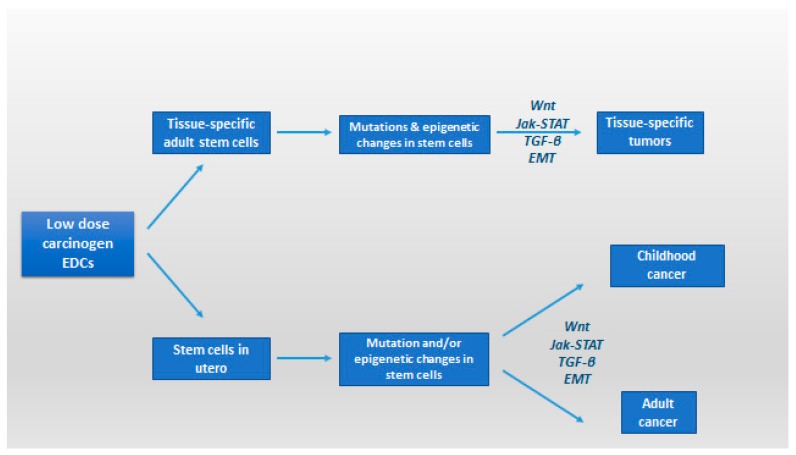
Regulation of cancer stem cells and epithelial plasticity by endocrine disruptors in low dose; potential pathways leading to malignant transformation and involvement of cancer stem-like cells (modified from [130]). EMT: epithelial–mesenchymal transition; Jak-STAT: Janus kinase/signal transducers and activators of transcription pathway; TGF-β: transforming growth factor-beta; Wnt: Wnt protein signaling pathways.

Endocrine disruptors may also affect the oncogenic transformation process, via reprogramming and remodeling of carcinogenic gene expression. Simultaneous with downregulation of E-cadherin is the epithelial–mesenchymal transition (EMT) [126]. The abnormal expression of E-cadherin has been detected in many types of cancers including gastric carcinomas, its reduced expression being related to tumor invasive growth and metastatic ability, hence the implication of EDs in loss of E-cadherin-mediated cell–cell adhesion is implicated in tumor cell invasion and metastasis formation [127]. Exposure in low doses to several environmental carcinogens might hasten this transition on the transcription factors that regulate EMT [127]. This procedure is also hastened by chronic inflammation mediated by NF-κB. During EMT procedure, an amount of inflammatory cells are attracted to the growing tumor mass [128]. Other factors may also be involved, such as low-dose environmental contaminants that regulate NF-κB transcription deteriorating the procedure [128,129]. From the practical point of view, it appears that human cells are predisposed to low-dose, short or chronic, exposure to EDs- induced malignant transformation and that epigenetic changes occurring in utero or in tissue-specific adult stem cells may have a future role in the development of cancer stem-like cells and associated tissue-specific cancers (Figure 3) [130].

### 3.6. Endocrine Disruptors Effect in Female and Male Reproductive Function

Endocrine Disruptors affect female reproductive health impairing reproductive organ function and promoting infertility. Organochlorines (OCs) for example regulate gene expression by interacting with the promoter regions of various genes such as *ERα* and *ERβ* [131], *AP-1* [132], *CYPs*, human pregnane X receptor, NF-κB [133], EGF [134], SP-1 [135], JunB, Fra-1, Fra-2 [135], cyclin D1 [136], cAMP, PKA, and p38MAPK [137]. Organochlorines through inhibition of adenylate cyclase, hypermethylation in syncytin-1 promoter and down-regulation of the OASIS and GCMa mRNA transcripts through epigenetic alterations may play important role in preeclampsia manifestation. Direct effects of estrogenic EDs [138] on female reproductive health [139] include hindered reproductive organ function [140], and infertility [141]. Similarly, ovarian dysfunction can result in decreased serum estradiol levels associated with sexual dysfunction [142]. Moreover, effects on the oocytes can possibly incite multigenerational impacts. The most severe and long-lasting ovarian, mammary and uterine diseases that occur in adulthood [143] are induced by exposures in fetal and neonatal periods [144]. Toxicity of OCs may deteriorate placental function and reignite developmental issues in the offspring. Chronic effects of OCs on trophoblast cells and later until cell differentiation might be the genomic alterations on expression or number of Ca^2+^-ATPase and/or second messengers [145]. Placental exposure to PCBs [146] has been demonstrated to affect maternal vasculature and produce degenerative alterations in the trophoblast and fetal vessels, resulting in fetal growth impairment or death [147].

Endocrine disruptors effects on male reproductive health present a respective pattern. Cryptorchidism and hypospadias are the commonest congenital malformations of newborn boys; testicular germ cell tumors are the commonest neoplasms in young adults; and prostate cancer is the overall leading cancer in older men. Around 15% of Western couples present fertility problems, with a contributory/sole male factor in at least half of the cases. A hypothesis that a common cause underlies these pathologies has been expressed but remains still controversial [148]. Based on epidemiological, clinical, biological and experimental evidence, it has been hypothesized that cryptorchidism, hypospadias, testicular cancer, and poor spermatogenesis are signs of a sole developmental disturbance, named testicular dysgenesis syndrome (TDS) [149,150]. Testicular dysgenesis syndrome (TDS) is considered result of embryonal programming/gonadal development disruption during fetal life and may be increasingly common due to adverse environmental influences, mainly exposure to EDs [149,150]. A great amount of research has therefore been focused on the effect of environmental factors among others, on male reproductive parameters [151]. Endocrine disruptors effects on male individuals include compromised development of androgen-dependent sex organs due to impaired testosterone production as well as disruption of sperm motility [5] and fertilizing ability at adulthood [6]. Dichlorodiphenyldichloroethane (DDE) for example is a potent androgen antagonist [152] suppressing spermatogenic epithelium in humans [153]. Lindane increases LH, FSH, and decreases testosterone but also can accumulate in the testes, impairing germinal epithelium and Sertoli cell function [154]. Exposure to environmental estrogens (Chlordecone) increases the risk of prostate cancer [155]. Phthalates reduce testosterone causing to hypospadias, agenesis of the gubernacular cords/epididymis and testicular atrophy [156].

## 4. Conclusions

Exposure to isolated EDs or ED mixtures, even at low doses, especially during crucial window time periods, can impact endocrine system through hypothalamic-pituitary-gonad/thyroid/adrenal axes, genetic and/or epigenetic alterations, oxidative stress, inflammatory cytokines, biochemical pathways, transcriptional and/or transgenerational pathways, and extra- and intracellular signaling, contributing to the pathogenesis of many human diseases including obesity and affect decisively the morbidity and mortality. Uses of ED mixtures are in need of a new holistic approach taking into account other environmental pollutants, new methodologies and processes. Population residents are exposed to a cumulative and synergistic network of EDs that changes every day. Despite European Directive on Waste Electrical and Electronic Equipment [157] the total global production of novel brominated flame retardants is estimated at 100,000–180,000 tons per year [158]. New compounds are constantly entering the market, and it has been estimated that over 3000 commercial Per- and Polyfluoroalkyl Substances compounds are circulating on the global market [159]. Uses of ED mixtures are in need of a new holistic approach taking into account other environmental pollutants, new methodologies and processes. Suggestions for implementation of a new direction towards elimination of adverse consequences stemming from exposure to EDs include enhanced international collaboration in industrial level for the constraint of EDs use, a strong legal framework, and the complementary training of specialized scientists about the use and handling of EDs.

## Figures and Tables

**Table 1:** Mechanisms of sex hormone function disturbance by pesticides (modified from [11]).

Test Compound	Cell Proliferation	Estrogen Receptor Transactivation	Androgen Receptor Transactivation	Aromatase Activity
Dieldrin	**↑**	**↑**	**↓**	**–**
Endosulfan	**↑**	**↑**	**↓**	**↓**
Methiocarb	**↑**	**↑**	**↓**	**–**
Pirimicarb	**–**	**↑**	**–**	**↑**
Propamocarb	**–**	**↑**	**–**	**↑**
Fenarimol	**↑**	**↑**	**↓**	**↓**
Prochloraz	**↓**	**↓**	**↓**	**↓**

(↑) Increased response; (↓) decreased response; (–) no effect.

**Table 2:** Obesity, diabetes mellitus type 2 and lipid disorders induced by endocrine disruptors are involved in metabolic syndrome with a small (x), medium (xx) and large (xxx) qualitative correlation (modified from [61]).

Chemical	Obesity	Diabetes Mellitus Type 2	Lipid Disorders/Fatty Liver
Bisphenol A	xxx	xxx	xxx
Di(2-ethylhexyl)phthalate	xxx	xxx	xxx
Dichlorodiphenyltrichloroethane/ Dichlorodiphenyldichloroethylene	xxx	xx	x
Polybrominated diphenyl ether			x
Perfluorooctanoic acid	xx		xxx
Perfluorooctanesulfonic acid		x	xxx
Tributyltin	xxx	xxx	xxx
Air Pollution	xx	xxx	xxx
Polycyclic aromatic hydrocarbons			
Polychlorinated biphenyls	x	xxx	xxx
2,3,7,8-tetrachlorodibenzo-p-dioxin		xx	xxx
Atrazine		x	xx
Benzo(a)pyrene	x		xx

**Table 3:** Categorization of oxidative stress biomarkers according to endocrine disruptor type.

Biomarkers in Oxidative Stress											
EDs	*DNA*	*MDA*	*LPO*	*EROD*	*GST*	*GSH*	*SOD*	*ROS*	*Vit. C*	*Vit. E*	*CAT*
Organochlorines	+	+	+	+	+	–	–	+	–	–	–
Polychlorinated biphenyls	+	+	+	+	–	–	–	+	–	–	+
2,3,7,8-tetrachlorodibenzo-p-dioxin	+	+	+	+	–	–	–	+	–	–	–
Polycyclic aromatic hydrocarbons	+	+	+	+	+	–	–	+	–	–	+
Perfluorinated compound	+	+	+	+	–	–	+	+	–	–	+
Di(2-ethylhexyl) phthalate	+	+	+	non	–	–	+	+	–	–	+
Diethylstilbestrol	+	+	+	+	–	–	–	+	–	–	–
Bisphenol A	+	+	+	non	–	–	–	+	–	–1	–
Tributyltin	+	+	+	non	–	–	+	+	–	–	+
Short-chain chlorinated paraffins	non	?	+	non	+	?	?	+	?	?	?
Polychlorinated naphthalene	?	+	+	+	?	–	?	+	?	non	?

CAT: catalase; DNA: deoxyribonucleic acid damage; EDs: endocrine dysruptors; EROD: ethoxyresorufin-*O*-deethylase; GSH: glutathione; GST: glutathione S-transferase; LPO: lipid peroxidation; MD: malondialdehyde; ROS: reactive oxygen species; SOD: superoxide dismutase; Vit. C: vitamin C; Vit. E: vitamin E.
